# Data gaps in anthropogenically driven local‐scale species richness change studies across the Earth's terrestrial biomes

**DOI:** 10.1002/ece3.2004

**Published:** 2016-03-25

**Authors:** Grace E. P. Murphy, Tamara N. Romanuk

**Affiliations:** ^1^ Department of BiologyDalhousie UniversityHalifaxNova ScotiaCanada

**Keywords:** Biodiversity, biomes, data gaps, global change, species richness

## Abstract

There have been numerous attempts to synthesize the results of local‐scale biodiversity change studies, yet several geographic data gaps exist. These data gaps have hindered ecologist's ability to make strong conclusions about how local‐scale species richness is changing around the globe. Research on four of the major drivers of global change is unevenly distributed across the Earth's biomes. Here, we use a dataset of 638 anthropogenically driven species richness change studies to identify where data gaps exist across the Earth's terrestrial biomes based on land area, future change in drivers, and the impact of drivers on biodiversity, and make recommendations for where future studies should focus their efforts. Across all drivers of change, the temperate broadleaf and mixed forests and the tropical moist broadleaf forests are the best studied. The biome–driver combinations we have identified as most critical in terms of where local‐scale species richness change studies are lacking include the following: land‐use change studies in tropical and temperate coniferous forests, species invasion and nutrient addition studies in the boreal forest, and warming studies in the boreal forest and tropics. Gaining more information on the local‐scale effects of the specific human drivers of change in these biomes will allow for better predictions of how human activity impacts species richness around the globe.

## Introduction

It is well recognized that the Earth's biodiversity is undergoing significant change resulting from various types of human activity (Vitousek et al. 1997; Chapin et al. 2000; Sala et al. 2000; Butchart et al. [Link]; Barnosky et al. 2011). Studies that explore changes in species richness resulting from anthropogenic drivers of change are essential to understand how human activity affects biodiversity and have important implications for ecosystem management and policy formation. While a number of attempts to synthesize the data from local‐scale biodiversity change studies have been conducted, results have been mixed, with some studies reporting significant decreases in local‐scale species richness (Zvereva et al. 2008; Gerstner et al. 2014; Murphy and Romanuk 2014; Newbold et al. 2015), while others have found little to no change (Vellend et al. 2013; Dornelas et al. 2014; Supp and Ernest 2014). Interestingly, the syntheses that have found significant declines in species richness are those that have specifically focused on the effects of human drivers of change, while the syntheses that have found little change in species richness included a variety of locations ranging from disturbed to pristine. The significant changes that have been found in local‐scale species richness resulting from human activity highlight the need to further examine where on Earth these changes are occurring and if there are consistent patterns of change in certain biomes resulting from specific drivers. Each of these syntheses has also highlighted similar biases in terms of where local‐scale species richness change studies are being conducted, with the majority of studies taking place in North America and Europe. Unfortunately, these data gaps severely limit ecologist's ability to make predictions about where and why local‐scale biodiversity change is occurring around the globe.

Several studies have shown that geographic biases exist in the ecological literature (Butchart et al. [Link]; Pereira et al. 2010; Ahrends et al. 2011). A 2012 review of terrestrial ecological observations for a 5‐year period reported an over‐representation of studies in temperate biomes and protected areas (Martin et al. 2012). While the overall geographic distribution of ecological studies has been reviewed, geographic biases in local‐scale species richness change resulting from anthropogenic drivers have not been analyzed. Given the recent popularity in synthesizing the results of local‐scale species richness change studies to make predictions about how local‐scale biodiversity is changing around the globe, a more detailed analysis of the geographic biases that exist in the literature is necessary. In this study, we examine the geographic biases that exist in terrestrial local‐scale species richness change studies and provide information about where future local‐scale species richness change studies need to be conducted. We specifically focus on species richness change resulting from anthropogenic global change drivers (land‐use change, species invasions, nutrient addition, and warming). We examine how the geographic biases differ for these drivers across the Earth's terrestrial biomes and determine the data gaps that exist based on three circumstances: (1) the land area that the biome covers; (2) the future change that the drivers are projected to have in each biome; and (3) the impact that the drivers have on the biodiversity of each biome.

The sensitivity of a particular biome to a global change driver will depend on the unique set of features that define the biome, and thus, it is expected that the effects of various global change drivers will differ across the Earth's biomes (Sala et al. 2000). For example, the impacts of climate change on productivity have been shown to differ widely among the Earth's biomes (Silva and Anand 2013; Zhang et al. 2013). Various studies have assessed the magnitude of global change drivers across the Earth's biomes and have provided estimates of which biomes are most vulnerable to certain drivers. The projections of future change across biomes resulting from various global change drivers demonstrate that certain data gaps in local‐scale species richness change studies might be justified based on the level of threat of a particular driver in a specific biome. For example, estimates of land‐use change for the year 2050 predict large proportions of future land‐cover change in temperate broadleaf and mixed forests along with temperate and tropical coniferous forests and project a much lower proportion of change in the tundra and boreal forest biomes (Lee and Jetz 2008). Therefore, we would expect to find the majority of studies examining the effects of land‐use change on local‐scale species richness to have been conducted in temperate forests and tropical coniferous forests. Projections of net change in the number of 100 of the world's worst invasive alien species for the year 2100 (Bellard et al. 2013) estimate that the highest increase in invasive species will occur in temperate mixed forests. Therefore, an over‐representation of invasion studies in the temperate forest biomes is well justified. By comparing the data gaps in local‐scale species richness change studies with estimates of which biomes will be most and least affected by global change drivers, we aim to identify the most critical data gaps and make suggestions as to where future local‐scale species richness change studies need to be conducted to fill in these gaps.

Knowledge of how global change drivers will impact local‐scale species richness and how these impacts compare across biomes is dependent upon available information of these impacts across the Earth's biomes, for which the ecological literature is currently lacking. The findings from large‐scale syntheses of local‐scale species richness change will play crucial roles in how human‐impacted ecosystems are managed in the future. Unfortunately, predictions for how human activity will impact local‐scale biodiversity around the globe are seriously hindered by the data gaps that exist in the literature. It is necessary for future biodiversity research to focus on biodiversity change in biomes that are expected to experience significant impacts from a human driver of change and where data on that driver is lacking. Here, we use a dataset of 767 responses of change in species richness taken from 638 human‐mediated species richness change studies to determine where geographic data gaps exist across the various global change drivers, and discuss whether these biases are justified based on the varying levels of threat that the drivers pose to the biomes.

## Methods

### Selection criteria

Our dataset was compiled by searching the biological literature for studies that reported the effects of anthropogenic drivers of change on local‐scale species richness in a terrestrial habitat. Four anthropogenic drivers of change that have been identified as major drivers of current biodiversity change were included in the dataset: land‐use change, species invasions, nutrient addition, and warming (Vitousek et al. 1997). We performed a literature search using the ISI Web of Science database using the following search expressions: “biodiversity “OR “species richness” OR “community change” AND (“invasi* species” OR “habitat loss” OR “land use change” OR “climate change” OR “warm*” OR increas* temperature” OR “eutrophication” OR “nutrient add*”). We also searched for studies in the references of relevant meta‐analyses and syntheses. A final search of the literature was completed on 17 October 2015. We included studies that experimentally manipulated one of the four drivers of change or observational studies that compared an impacted site with a control (nonimpacted) site. The literature search yielded 638 suitable studies with 767 values of change in species richness that were included in the final dataset. Only studies conducted in terrestrial ecosystems were included in the dataset. This dataset specifically focuses on studies that examine change in species richness in control vs. impacted treatments. Studies that assessed other metrics like evenness and biodiversity indices, those that assessed changes in community composition, and those that examined species richness change using time‐series or before–after studies were not included in the dataset. We chose to focus only on species richness, as it is the most common biodiversity measure used in studies examining the impact of anthropogenic global change drivers.

Geographic coordinates were either taken from the papers or in cases where the coordinates were not given they were estimated using Google Earth (Google Inc., California, USA). Studies that reported multiple measures of species richness for the same geographic location (i.e., different species, driver intensities, etc.) were only included in the dataset once and the values were averaged when calculating effect sizes. We only included multiple responses from the same study when they were taken from different countries or biomes. The selection criterion is similar to that in Murphy and Romanuk (2014) and a more detailed explanation can be found there. For the purposes of examining data gaps, the habitat loss and land‐use change categories have been combined. Also, for the current study we only included invasion studies that give a measure of species richness change in the entire community rather than just native species.

### Data analysis

To investigate the data gaps existing across the Earth's biomes, we categorized study responses using the 14 global terrestrial ecoregions identified by Olson et al. (2001). We visualized the global distribution of studies by entering the locations of all 767 responses into ArcGIS 10.1 (ESRI 2011). We used chi‐squared tests to determine whether specific biome–driver combinations are over‐represented or under‐represented. We tested for significant differences between the observed and expected distribution of studies based on three sets of circumstances: (1) the relative land area that the biome covers (e.g., biomes with a larger land area are expected to contain a proportionately greater number of responses); (2) the future change that the drivers are projected to have in each biome (e.g., biomes projected to experience a larger increase in a driver are expected to contain a greater number of responses); and (3) the impact that the drivers have on biodiversity in each biome (e.g., biomes where a driver has a larger impact on biodiversity are expected to contain a greater number of responses). We used projections from various studies to determine the future change of drivers among biomes. For land‐use change, we used the projected proportions of land‐cover transformation due to land‐use change for the year 2050 given in Lee and Jetz (2008). Estimates are given for 57 biome–realm combinations and for four socioeconomic scenarios. We averaged the estimates for the biome–realm combinations and across the four socioeconomic scenarios to get one estimate of future land‐use change for each biome. Using these estimates, we determined the proportion of projected land‐use change for each biome for the year 2050 (Table 1). For species invasions, we used estimates of the percentage of net change in the numbers of 100 of the world's worst invasive species for the year 2080 given in Bellard et al. (2013). The biomes categorized in this study slightly differ from our categorization so in some cases the estimates from multiple biomes were averaged. As this study estimates that some biomes will become less suitable for invasive species (i.e., the tropics), the values given for percentage of net change are negative. To calculate the proportion of change in invasive species for each biome and the expected number of studies, we first added 5 to each estimate, thus making all estimates positive (Table 1). For nutrient addition, we used the expected changes for the year 2100 given in Sala et al. (2000). This study ranks the biomes from 1 to 5 based on future change in nitrogen deposition, and using these ranks, we calculated the proportion of change for each biome (Table 1). To determine the expected number of warming studies based on future change, we used the climate anomalies between pre‐industrial conditions and projections for the year 2100 given in Benito‐Garzon et al. (2014) (Table 1). To calculate the expected number of studies based on the impact that the drivers have on the biodiversity of each biome, we used the estimates given in Sala et al. (2000) where the impact of a large change in each driver on biodiversity is ranked from 1 to 5 for each biome. The proportion of impact that each driver has on each biomes biodiversity was calculated from these rankings (Table 1).

**Table 1:**
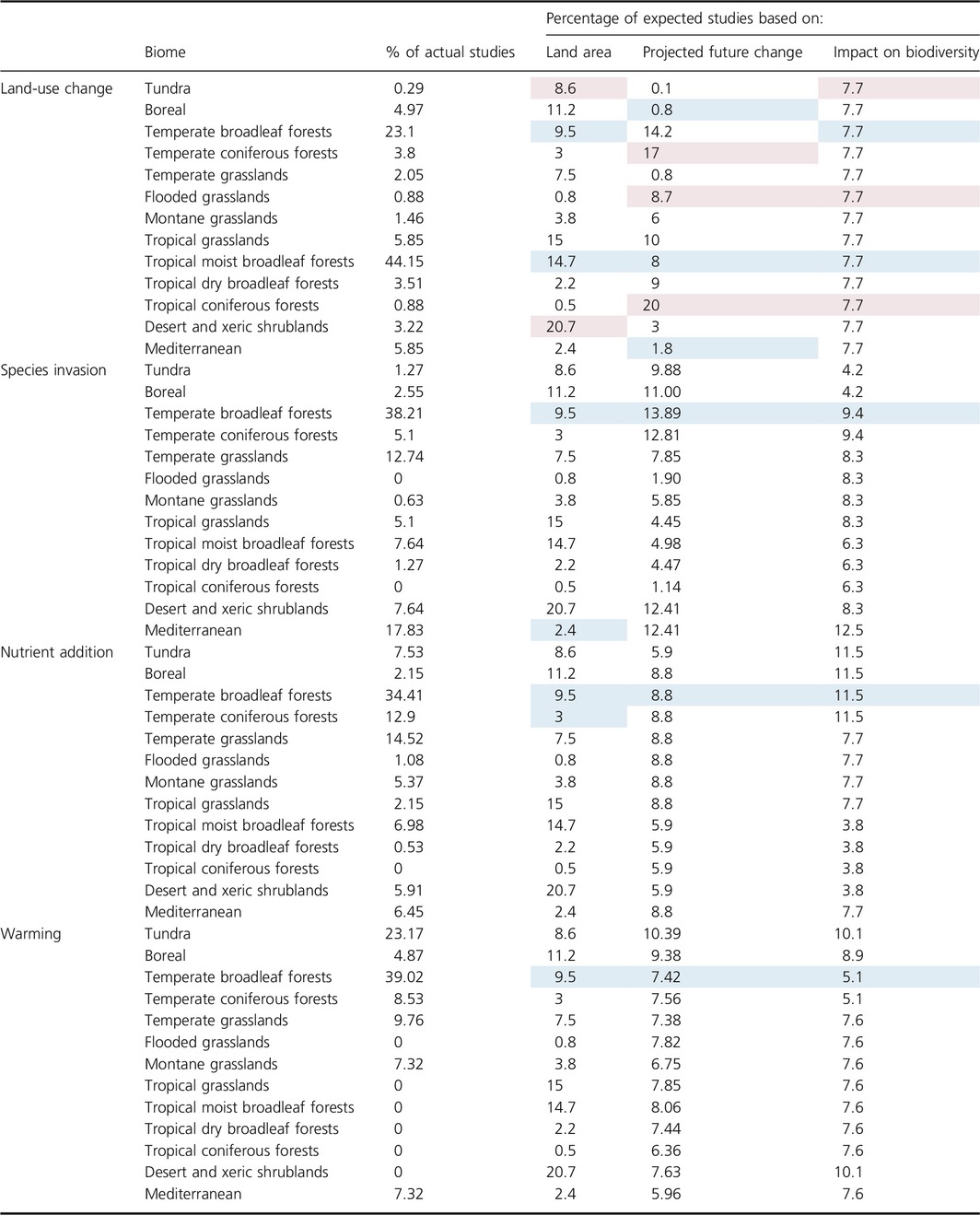
Percentage of anthropogenically driven local‐scale species richness change studies conducted in each biome for four drivers of change, along with the expected percentage of studies based on the land area covered by each biome, the projected future change, and the impact of the driver on biodiversity. Values highlighted indicate that the biome–driver combination is significantly over‐ (blue) or under‐ (red) represented (*P* < 0.05) based on the highlighted circumstance

We conducted a separate chi‐squared test for each driver of change and used the total number of studies for each driver to calculate the expected number of studies. For example, the total number of land‐use change studies across all biomes is 342; therefore, to calculate the expected number of studies in each biome for the chi‐squared test based on land area, we multiplied the proportion of land area that each biome covers by 342 to give the expected values. These values were then compared to the observed number of studies in each biome in the chi‐squared test. Biome–driver combinations were considered significantly over‐ or under‐represented if the *P*‐value was equal to or less than 0.05.

We used the standard deviation of the average effect sizes for each biome–driver combination as a measure of how variable the magnitude of change in species richness is in each biome. The effect size compares species richness between the control (*c*) and impacted (*i*) treatments in each study and was calculated as the response ratio (Hedges et al. 1999):

$$\text{RR}=\ln\left(\frac{{\overset{¯}{X}}_{i}}{{\overset{¯}{X}}_{c}}\right)$$

## Results

### Global study distribution

Our results reveal several biases in the global distribution of anthropogenically driven, local‐scale species richness change studies. Studies included in the dataset were conducted in 79 countries. The five countries with the greatest number of responses were as follows: USA (23.6% of responses), China (8.1% of responses), Brazil (6.5% of responses), Canada (5% of responses), and Australia (3.7% of responses). The Palearctic and Nearctic are the most commonly studied biogeographic realms with 33.8% and 28.6% of studies conducted in these realms, respectively. Across all studies, the Afrotropic and Antarctic realms are significantly under‐represented based on land area (*P* < 0.001 and *P* = 0.021, df = 7, respectively), while the Nearctic and Oceanic realms are significantly over‐represented (*P* < 0.001, df = 7). Based on land area, land‐use change studies are significantly over‐represented in the Neotropics (*P* < 0.001, df = 7) and are significantly under‐represented in the Palearctic (*P* = 0.001, df = 7). Both species invasion and nutrient addition studies are significantly under‐represented in the Afrotropics (*P* = 0.014 and *P* < 0.001, df = 7, respectively) and significantly over‐represented in the Nearctic and Oceanic (*P* < 0.001, df = 7) realms. Warming studies are significantly over‐represented in the Nearctic realm (*P* < 0.001, df = 7).

### Study distribution by biome

**Figure 1:**
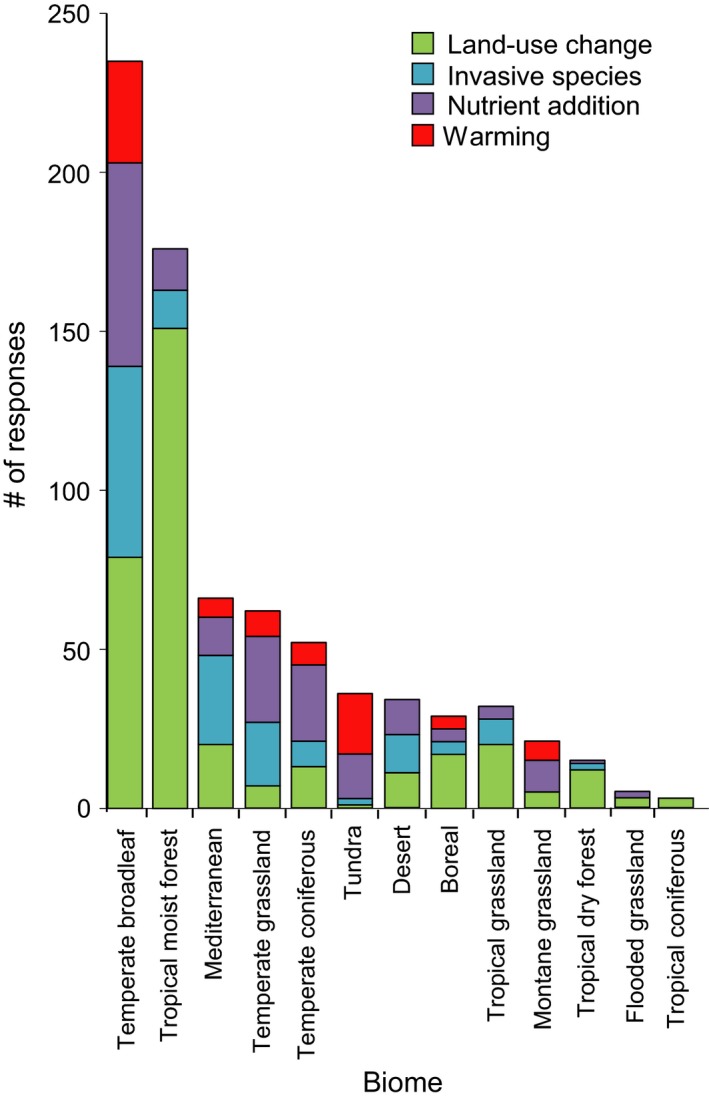
Number of local‐scale species richness change study responses across 13 terrestrial biomes and four human drivers of change.

**Figure 2:**
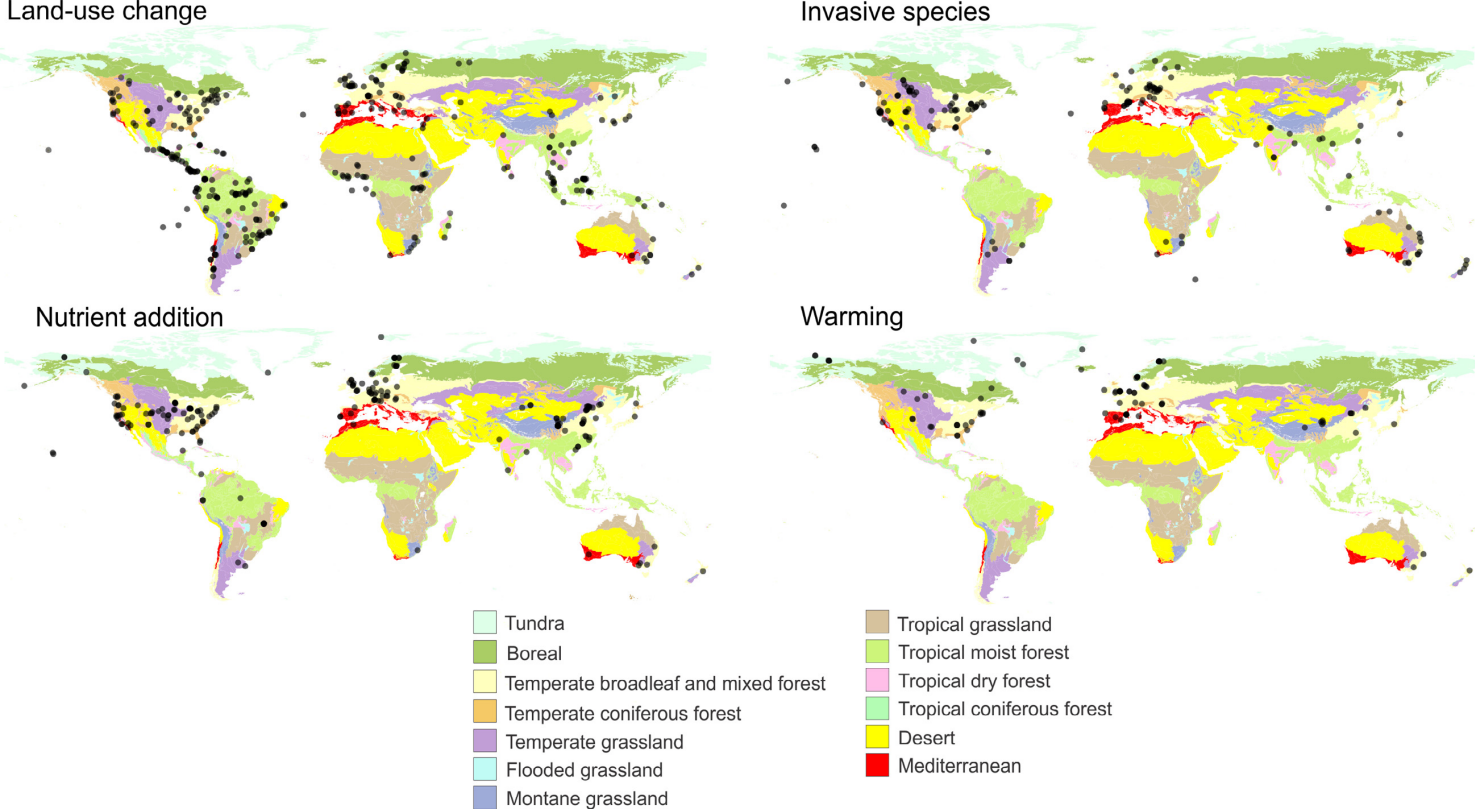
Maps of the global distribution of local‐scale species richness change studies for each of the four human drivers of change overlaid on the distribution of the 13 terrestrial biomes used in the analysis (Olson et al. 2001). Points are semitransparent with darker areas indicating an overlap of studies.

Across all drivers, temperate broadleaf and mixed forests (31%) and tropical moist broadleaf forests (23%) are the most frequently studied biomes (Figs. 1, 2). All other biomes contain fewer than 10% of responses. Based on land area, the boreal forest and tropical grassland biomes are significantly understudied (*P* < 0.001, df = 12), while the temperate broadleaf and mixed forest, temperate coniferous forest, tropical moist broadleaf forest and Mediterranean biomes are significantly over‐represented (*P* < 0.001, df = 12).

There is notable disparity in how frequently studied a biome is when the four drivers of change are examined separately (Figs. 1, 2). One of the most significant areas of bias in our analysis of human driven local‐scale species richness change studies is the over‐representation of land‐use change studies in tropical and temperate broadleaf forest biomes. Almost half of the land‐use change studies are conducted in tropical moist broadleaf forests and almost a quarter of land‐use change studies are conducted in temperate broadleaf and mixed forests. Based on the proportion of land area that these biomes cover, they are significantly over‐represented, with three times the number of expected land‐use change responses in the tropical moist broadleaf forest (*P* < 0.001, df = 12), and over two times, the number of expected responses in the temperate broadleaf and mixed forest (*P* < 0.001, df = 12). All other biomes contain less than 6% of the land‐use change studies (Fig. 1), and based on land area, the tundra and desert biomes are under‐represented (*P* = 0.007 and *P* < 0.001, df = 12, respectively). When analyzed based on the projection of future land‐use change and the impact that land‐use change has on the biodiversity of each biome, the tropical moist broadleaf forest remains over‐represented in both categories (*P* < 0.001, df = 12). The temperate broadleaf and mixed forest biome is over‐represented based on the impact on biodiversity (*P* < 0.001, df = 12), but not based on projected future land‐use change. Both the temperate and tropical coniferous forest biomes are significantly under‐represented based on the magnitude of land‐use change expected to occur in these biomes in the future (*P* < 0.001, df = 12), and the tropical coniferous forest is almost significantly under‐represented based on the impact to biodiversity (*P* = 0.055, df = 12). The temperate grassland and tropical dry forest biomes have the lowest variance in the magnitude of species richness change following land‐use change (SD = 0.149, *n* = 7 and SD = 0.2, *n* = 12, respectively), while the desert and tropical coniferous forest biomes have the highest variance (SD = 0.793, *n* = 11 and SD = 0.869, *n* = 3, respectively) (Figure S1).

Studies that examine the effect of species invasions on local‐scale species richness change are significantly over‐represented in the temperate broadleaf and mixed forests (p < 0.001, df = 12; 38% of studies) based on all three categories (Table 1). Species invasion studies are also over‐represented in the Mediterranean biome based on land area (*P* < 0.001, df = 12; 18%), but not based on the projected extent of exotic invasions and the impact of invaders on Mediterranean biodiversity. Invasion studies are relatively well represented in temperate grasslands, deserts, and tropical moist broadleaf forests, yet all other terrestrial biomes contain 5% or fewer of the invasive species responses (Table 1). The tundra and boreal forest biomes have the lowest variance in the magnitude of species richness change following species invasions (SD = 0.021, *n* = 2 and SD = 0.085, *n* = 4, respectively), while the temperate broadleaf forest (SD = 0.558, *n* = 60), temperate grassland (SD = 0.606, *n* = 20), and Mediterranean (SD = 0.559, *n* = 28) biomes have the highest variance (Figure S1).

Over half of nutrient addition studies are conducted in the temperate region (Fig. 1, Table 1). Based on land area, nutrient addition studies are significantly over‐represented in temperate broadleaf and temperate coniferous forests (*P* < 0.001, df = 12), but temperate broadleaf and mixed forests are the only biome that is significantly over‐represented based on the projected levels of future nutrient addition and the impact on biodiversity (Table 1). Nutrient addition studies are well represented in the tundra, yet fewer are conducted in the boreal forest (Table 1). The flooded grassland (SD = 0.046, *n* = 2), temperate grassland (SD = 0.151, *n* = 27), and tundra (SD = 0.159, *n* = 14) biomes have the lowest variance in the magnitude of species richness change following nutrient addition, while the tropical moist forest (SD = 0.928, *n* = 13) and boreal forest (SD = 0.474, *n* = 4) biomes have the highest variance (Figure S1).

Similar to the other drivers, warming studies are over‐represented in temperate broadleaf and mixed forests based on land area, projections of future change, and impact on biodiversity (*P* < 0.001, df = 12; 39% of studies). Warming studies are also well represented in the tundra biome (23%), yet are lacking in the boreal forest (5%) and nonexistent in the tropics with zero warming studies conducted in tropical grasslands and the three tropical forest biomes (Fig. 1, Table 1). The temperate grassland (SD = 0.142, *n* = 8) and temperate broadleaf forest (SD = 0.17, *n* = 32) biomes have the lowest variance in the magnitude of species richness change following warming, while the montane grassland (SD = 0.53, *n* = 6) and tundra (SD = 0.564, *n* = 19) biomes have the highest variance (Figure S1).

## Discussion

It is evident that research intensity in the various terrestrial biomes is skewed toward different anthropogenic drivers of change. Below we discuss the differences in research intensity across biomes for each of the four human drivers of change and identify the most critical data gaps in terms of where future human driven local‐scale species richness change studies need to be conducted.

### Land‐use change

The majority of land‐use change studies are conducted in tropical moist forests and temperate broadleaf forests. It is not surprising that land‐use change studies are most common in these biomes as habitat loss due to human activity and land conversion has been most extensive in tropical and temperate forests (Miles et al. 2006; Potapov et al. 2009). Estimates of land‐use change for the year 2050 project an 11.5% change in land cover in tropical moist forests and a 19.5% change in tropical broadleaf and mixed forests worldwide (Lee and Jetz 2008). While the projections of future land‐cover change in these two biomes are large, there are other biomes with higher proportions of projected land‐cover change that are much less studied. These include temperate coniferous and tropical coniferous forests, which are projected to experience 23% and 28% land‐cover change, respectively, by the year 2050 (Lee and Jetz 2008), yet very few land‐use change studies that examine changes in local‐scale species richness are conducted in these biomes. This lack of studies is particularly concerning for tropical coniferous forests as not only is this biome significantly under‐represented based on both projections for future change and the impact of change on biodiversity but tropical coniferous forests also have the highest variance in the magnitude of local‐scale species richness change following land‐use change of all the biomes (Figure S1). Compared with the other biomes, the variance in the magnitude of local‐scale species richness change following land‐use change is also relatively high in the tropical moist forests so the large number of land‐use change studies conducted in this biome is justified (Figure S1). However, given the significant impact that land‐use change has on biodiversity (Newbold et al. 2015), shifting some of the focus of future land‐use change studies from tropical moist and temperate broadleaf forests to the coniferous forest biomes would fill crucial data gaps in how land‐use change impacts biodiversity around the globe.

### Invasive species

Studies that examine the effect of species invasions on local‐scale species richness are most commonly conducted in temperate broadleaf forests (38% of responses) and the Mediterranean (18% of responses). Invasions by exotic species are most likely in regions of high human activity (Didham et al. 2005; MacDougall and Turkington 2005) such as the Mediterranean, temperate forest, and grassland biomes, as opposed to remote areas, such as the tundra and boreal forest (Sala et al. 2000). The over‐representation of invasion studies in the temperate broadleaf and mixed forest biome is well justified as it has been estimated that temperate mixed forests will experience a high increase in invasive alien species for the year 2100 (Bellard et al. 2013). Our results also show that the temperate broadleaf forest and Mediterranean biomes have high variance in the magnitude of change in local species richness following species invasions so while these biomes are significantly over‐represented further studies in these biomes will be useful in deciphering this variability (Figure S1).

We found relatively few species invasion studies conducted in the tropical biomes (8% in tropical moist forests, 5% in tropical grasslands, 1% in tropical dry forests, and none in tropical coniferous forests) compared with the large number of total studies conducted in this biome. Species invasions are not thought to be one of the major threats to biodiversity in the tropical biomes as the high diversity in the tropics minimizes the chance of non‐native species successfully invading (Sala et al. 2000). Also, as temperatures increase fewer species will be able to successfully inhabit the tropics. Bellard et al. (2013) predict that the shift of tropical forests to more extreme climates compared with higher latitude biomes will lead to the tropics becoming less suitable for invasive species in the future. Therefore, the distribution of invasion studies throughout the tropical biomes is appropriate to the level of threat that invasions will have in these areas in the future.

Less than 3% of the invasion studies in our dataset are conducted in the tundra and boreal forest biomes. These biomes also have the lowest variation in the magnitude of change in local species richness following invasions compared with the other biomes. Therefore, the few studies conducted in the tundra and boreal forest all show very similar change in species richness from species invasions, suggesting that the low number of studies in these biomes is justified. However, increasing temperatures are resulting in dramatic northward species range shifts (Chen et al. 2011) and warmer temperatures and longer growing seasons are enabling the survival of more non‐native species to high‐latitude ecosystems (Carlson and Shephard 2007; Spellman et al. 2014). The ecological effects of the increasing number of exotic species inhabiting the boreal forest are poorly understood (Sanderson et al. 2012). The boreal forest plays a key role in various processes that are crucial for global ecosystem functioning, such as biogeochemical cycling (Volney and Fleming 2000), and given the detrimental effects of invasive species on native biodiversity and ecosystem functioning (Vitousek et al. 1996; Murphy and Romanuk 2014), additional studies of the effects of species invasions on boreal forest biodiversity would be useful.

### Nutrient addition

The addition of excess nutrients to ecosystems is expected to be most prevalent in regions of high industrialization and intense agricultural activity (Galloway et al. 2004). Studies that examine the effects of nutrient addition on local‐scale species richness are most common in temperate broadleaf and mixed forests (34% of responses). Furthermore, while studies of the three other human drivers of change are lacking in the temperate coniferous forest, there are a relatively high number of nutrient addition studies conducted in this biome (13% of responses). The temperate regions of the Earth have large population density and increasing agricultural activity (Sala et al. 2000). Nutrient addition is expected to be a major driver of future biodiversity change in the temperate forest biomes; therefore, the over‐representation of studies in the temperate forests is not surprising. The variance in the magnitude of change in local species richness following nutrient addition is also relatively high in both temperate forest biomes, which further justifies the over‐representation. Nutrient addition studies are disproportionately conducted in temperate forest and tundra biomes and understudied in the boreal forest biome (2% of responses). Nutrient addition is not expected to have as large an impact on biodiversity in the tropical forest and desert biomes as plant growth in these regions is primarily limited by water availability rather than nitrogen (Vitousek 1984), yet we found more nutrient addition studies conducted in these biomes (7% and 6% of responses, respectively) than in the boreal forest. Like the tundra biome, nutrient addition in the boreal forest is not generally acknowledged as an immediate threat due to its distance from sources of pollution (Sala et al. 2000). However, atmospheric nitrogen deposition from agricultural and industrial activity is currently increasing and has the potential to be transported long ranges, resulting in impacts on remote ecosystems (Bergstrom and Jansson 2006; Holtgrieve et al. 2011). The boreal forest is an example of a relatively pristine ecosystem that could suffer immense consequences from increased atmospheric nitrogen deposition, as boreal forest plant species are nitrogen limited. Furthermore, the high variance in the magnitude of change in species richness following nutrient addition in the boreal forest suggests that more studies are necessary to determine how boreal forest biodiversity responds to nutrient addition.

### Warming

Warming studies are most common in temperate broadleaf and mixed forests (39% of responses) and are better represented in the tundra biome compared with the other four drivers of change (23% of responses). Global circulation models predict larger increases in temperature at higher latitudes (Kattenberg et al. 1996); thus it is not surprising that the majority of studies examining the effects of increasing temperatures on species richness are conducted in these high‐latitude biomes. Interestingly, the magnitude of change in species richness is highly variable in the tundra biome and is much less variable in the temperate broadleaf and mixed forests (Figure S1). This suggests that future studies should focus more on the impacts of warming on tundra biodiversity than on temperate broadleaf forest biodiversity, which is the opposite to what is currently occurring.

Only 5% of warming studies are conducted in the boreal forest yet, given its high latitude, this biome is expected to experience significant increases in temperature compared with other biomes. None of the warming studies included in our dataset are conducted in tropical grassland, tropical forests, and desert biomes. While these lower latitude biomes are not expected to experience as large an increase in temperature compared with higher latitude biomes, such as the boreal forest and tundra (Sala et al. 2000), the ecological communities present in these biomes are expected to be less robust than species at higher latitudes to the effects of warming (Sunday et al. 2011). It has been suggested that the lower thermal tolerance of tropical species will result in increasing temperatures to have more detrimental effects on tropical communities (Tewksbury et al. 2008; Tuck and Romanuk 2012). The difference in the effects of climate change along latitudinal gradients highlights the need to conduct studies and collect data on the effects of warming on species richness across all of the Earth's biomes, not just those where temperatures are expected to increase the most.

## Conclusions

In this study, we identify terrestrial biomes where studies that examine the effects of certain human drivers on local species richness are severely lacking based on the level of threat that the drivers pose to the biomes. The biome–driver combinations that are most critical in terms of where future biodiversity change studies need to be conducted are as follows: land‐use change studies in temperate and tropical coniferous forests, species invasion, and nutrient addition studies in the boreal forest, and warming studies in the boreal forest and tropical biomes.

An important caveat to our identification of data gaps is that we only consider studies that examine the effects of human drivers on species richness in control vs. disturbed treatments. There are a variety of other biodiversity change studies that were not included in this analysis. We chose to focus only on species richness, as it is the most common biodiversity measure used in studies examining the effects of human drivers of ecosystem change. However, we expect roughly similar data gaps and patterns across biomes for studies that use biodiversity metrics other than species richness.

## Conflict of Interest

None declared.

## Supporting information


**Figure S1**. Variance (given as standard deviation) in the magnitude of change in species richness following the four human drivers of change.
