# Supplementary figures and images for: Data gaps in anthropogenically driven local‐scale species richness change studies across the Earth's terrestrial biomes
Source: Ecol Evol. 2016 Mar 25;6(9):2938–47. doi: 10.1002/ece3.2004 (PMC4808076; doi:10.1002/ece3.2004)

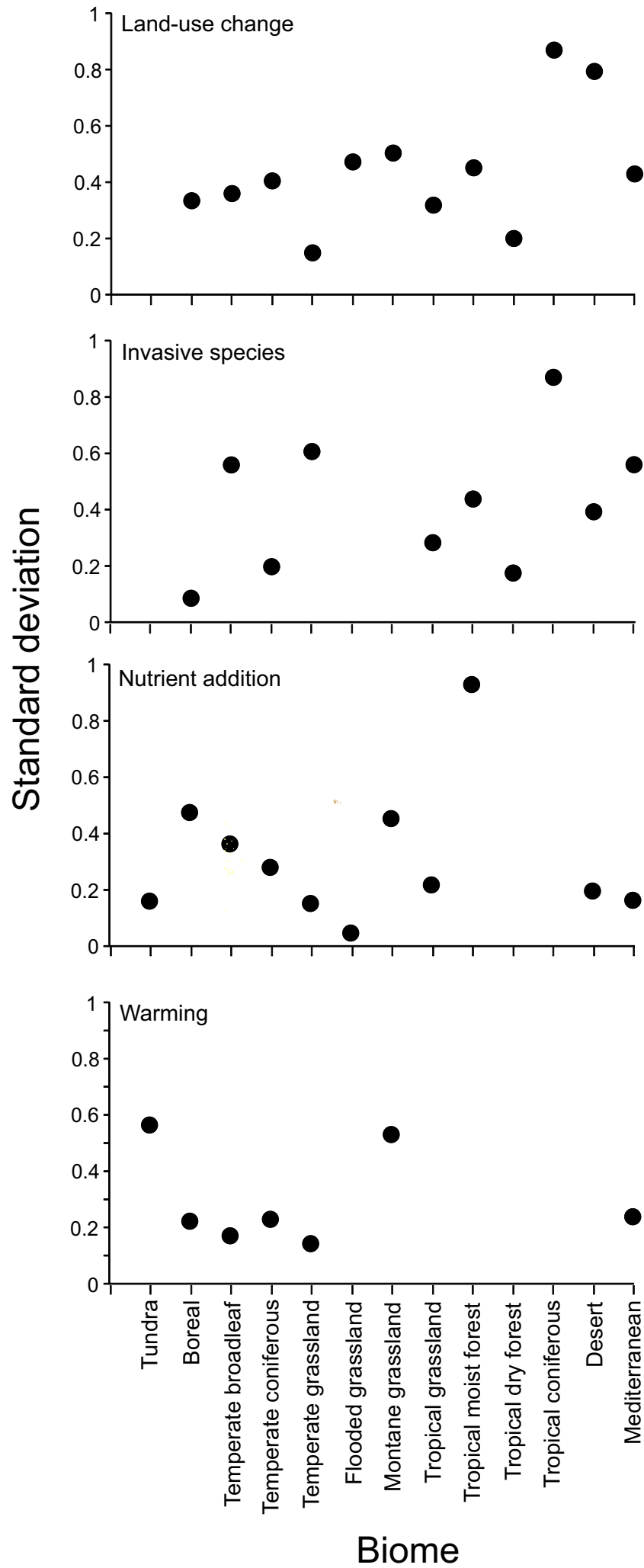

Supplement: Supplementary file 1 — Figure S1. Variance (given as standard deviation) in the magnitude of change in species richness following the four human drivers of change. [file ECE3-6-2938-s001.pdf]
